# A Novel Pyroptosis-Related Gene Signature for Predicting the Prognosis and the Associated Immune Infiltration in Colon Adenocarcinoma

**DOI:** 10.3389/fonc.2022.904464

**Published:** 2022-07-14

**Authors:** Zhiyuan Chen, Zheng Han, Han Nan, Jianing Fan, Jingfei Zhan, Yu Zhang, He Zhu, Yu Cao, Xian Shen, Xiangyang Xue, Kezhi Lin

**Affiliations:** ^1^ Wenzhou Collaborative Innovation Center of Gastrointestinal Cancer in Basic Research and Precision Medicine, Wenzhou Key Laboratory of Cancer-Related Pathogens and Immunity, Department of Microbiology and Immunology, Institute of Molecular Virology and Immunology, School of Basic Medical Sciences, Wenzhou Medical University, Wenzhou, China; ^2^ Department of General Surgery, The Second Affiliated Hospital and Yuying Children's Hospital of Wenzhou Medical University, Wenzhou, China; ^3^ School & Hospital of Stomatology, Wenzhou Medical University, Wenzhou, China; ^4^ School of Second Clinical Medical, Wenzhou Medical University, Wenzhou, China; ^5^ Department of General Surgery, The First Affiliated Hospital of Wenzhou Medical University, Wenzhou, China; ^6^ Experimental Center of Basic Medicine, Wenzhou Medical University, Wenzhou, China

**Keywords:** COAD, pyroptosis, gene signature, prognostic model, tumor microenvironment, tumor immunity

## Abstract

**Background:**

Pyroptosis has been demonstrated to be an inflammatory form of programmed cell death recently. However, the expression of pyroptosis-related genes (PRGs) in colon adenocarcinoma (COAD) and their correlations with prognosis remain unclear.

**Methods:**

Data of COAD patients were obtained from The Cancer Genome Atlas (TCGA) database to screen differentially expressed genes (DEGs). Univariate Cox regression analysis and the LASSO Cox regression analysis were applied to construct a gene signature. All COAD patients in TCGA cohort were separated into low-risk subgroup or high-risk subgroup *via* the risk score. Kaplan–Meier survival analysis and receiver operator characteristic (ROC) curves were adopted to assess its prognostic efficiency. COAD data from the GSE17537 datasets was used for validation. A prognostic nomogram was established to predict individual survival. The correlation between PRGs and immune cell infiltration in COAD was verified based on TIMER database. CIBERSORT analysis was utilized on risk subgroup as defined by model. The protein and mRNA expression level of PRGs were verified by HPA database and qPCR.

**Results:**

A total of 51 differentially expressed PRGs were identified in TCGA cohort. Through univariate Cox regression analysis and LASSO Cox regression analysis, a prognostic model containing 7 PRGs was constructed. Kaplan–Meier survival analysis indicated that patients in the low-risk subgroup exhibited better prognosis compared to those in the high-risk subgroup. Additionally, the area under the curve (AUC) of ROC is 0.60, 0.63, and 0.73 for 1-, 3-, and 5-year survival in TCGA cohort and 0.63, 0.65, and 0.64 in validation set. TIMER database showed a strong correlation between 7 PRGs and tumor microenvironment in COAD. Moreover, CIBERSORT showed significant differences in the infiltration of plasma cells, M0 macrophages, resting dendritic cells, and eosinophils between low-risk subgroup and high-risk subgroup. HPA database showed that protein expression level of SDHB, GZMA, BTK, EEF2K, and NR1H2 was higher in normal tissues. And the transcriptional level of CASP5, BTK, SDHB, GZMA, and RIPK3 was high in normal tissues.

**Conclusions:**

Our study identified a novel PRGs signature that could be used to predict the prognosis of COAD patients, which might provide a new therapeutic target for the treatment of COAD patients.

## Introduction

As the third most common cancer in the world, colorectal cancer (CRC) is the fourth leading cause of cancer-related death worldwide (1). Fortunately, total CRC mortality is decreased by early detection and modified treatment methods. However, the 5-year survival rate of patients with metastatic CRC is thought to be less than one in seven, depending on differences in disease and treatment strategies (2). It is predicted that over 2.2 million new cases and 1.1 million deaths will take place by 2030 (3, 4). It is widely known that the improvement of individualized treatment requires the refinement of subtypes of cancer according to their histological and genetic characteristics. Among all the subtypes, colon adenocarcinoma (COAD) is the most common histological subtype, accounting for more than 90% of CRC (5).

In recent years, the correlation between tumor biological characteristics and gene expression of COAD has been widely reported through the exploration of public database. Dong et al. reported that MYC and KLK6 were highly expressed and significantly associated with overall survival in patients with COAD. The results of drugbank showed that MYC and KLK6 were associated with a variety of medications (6). Liu et al. constructed invasion-related 6-gene signature, and Western blot analysis and immunohistochemistry were performed to validate protein expression (7). Jiang et al. constructed a co-expression network of differential expressed genes (DEGs) and survival-related genes in TCGA-COAD, and a prognostic prediction system based on a 65−gene signature was established using this co−expression network, which showed a good prediction effect (8). Bao et al. built a robust microsatellite status-related gene signature to predict the prognosis and differentiate between microsatellite instability(MSI) and microsatellite stability(MSS) tumors (9). Zhu et al. reported a methylation-driven genes (MDGs)-related risk prediction model and confirmed that the mRNA expression levels of MDGs were regulated by the methylation of their promoter regions (10).

Pyroptosis, which is known as cellular inflammatory necrosis, is regarded as gasdermin-mediated inflammatory death (11). There appears DNA damage and chromatin condensation in pyroptosis, which is similar to apoptosis (12, 13). Besides, pyroptotic cells swell with a lot of bubble-like protrusions on the membrane surface before membranolysis (14). Different from the explosive rupture in necrosis, plasma membrane leakage in pyroptosis only flattens the cytoplasm (14). The reason behind this phenomenon might be that caspases activation or release of granzymes results in the N-terminal of gasdermin oligomerization and pore formation (1–2 μm in diameter) in the plasma membrane, which allows mature IL-1β/IL-18 with a diameter of 4.5 nm and caspase-1 with a diameter of 7.5 nm to pass through, respectively (15). Contemporaneously, cell swelling and osmotic lysis are caused by the water that enters through the pores, which leads to membranolysis and the release of IL-1β and IL-18 (16).

Recently, an increasing number of studies indicate that pyroptosis has a close relationship with the development of tumors. In a study, it is shown that the expression of NALP1 decreased in colon cancer tissue compared with normal tissues (17). On the contrary, the occurrence of colon cancer is inhibited by DCA, which could restore the expression of NALP1, indicating that NALP1 is a potential therapeutic marker for colorectal carcinoma (17, 18). Additionally, accompanied with the release of substances within the cell, pyroptosis is inevitably related to regulation of the tumor microenvironment. The defect in GSDMD alleviated the cytolytic ability of CD8+ T cells, suggesting that GSDMD is essential in the immune response of tumor cells (19). However, the prognostic value of pyroptosis-related genes (PRGs) in COAD has not yet been elucidated.

In the present research, the PRG expression profiles and the prognostic capacity in COAD are investigated through bioinformatics analysis. Our research may witness the discovery of new prognostic biomarkers and therapeutic targets in COAD.

## Materials and methods

### Datasets and Acquisition

Fragments per kilobase million (FPKM) normalized expression profile data of 437 samples were derived from The Cancer Genome Atlas (TCGA) database by GDC data transfer tool and merged into an expression matrix, including 398 colon adenocarcinoma (COAD) and 39 normal colon samples. According to human gene annotations (Homo_sapiens.GRCh38.101.CRH.GTF), the Ensemble IDs were transformed into gene symbols. Then, the clinical data of COAD patients were downloaded and combined into another matrix. Excluding the patients whose follow-up duration and recorded date of death is incomplete, 368 COAD patients remained. Additionally, raw data (Counts) of 421 samples (382 COAD and 39 normal colon samples) were gained from TCGA to differentially expressed gene (DEG) analysis. Moreover, somatic datasets for COAD were also downloaded from TCGA. Additionally, a gene expression omnibus (GEO) dataset, GSE17537, which contained the microarray-based of 55 COAD patients and corresponding clinical data respectively, were downloaded from GEO website. GSE17537 used the GPL570 platform. With R package “Rsubread” and GPL570 annotation file, read count summaries were constructed to convert the probe IDs into gene symbols. One hundred thirty three pyroptosis-related genes (PRGs) were downloaded in the GeneCards database (https://www.genecards.org/).

### Identification of PRGs Between COAD and Normal Tissues

Intersected with 133 PRGs, a total of 51 PRGs were identified as DEGs by R package “limma” with a threshold of |log2FC| > 1 and false discovery rate (FDR) < 0.05. The upregulating or downregulating situation of 51 PRGs was shown in heatmap by R package “pheatmap“.

### Overview of Genetic Variation of 51 PRGs

The mutation frequency and oncoplot or waterfall plot of 51 PRGs in COAD patients were produced by R package “maftools”.

### Functional Enrichment Analysis of 51 PRGs

Gene Ontology (GO) enrichment and Kyoto Encyclopedia of Genes and Genomes (KEGG) pathway analysis were performed utilizing R package “clusterProfiler” based on 51 PRGs, with the criteria of FDR < 0.05. Both of them were described by R package “ggplot2”.

### Development of a Pyroptosis-Related Prognostic Gene Model

Cox regression analysis was employed to evaluate the correlations between 51 PRGs and survival status in the TCGA cohort, respectively, with a cutoff of *P* = 0.2 (20). Subsequently, 11 PRGs were clarified for further analysis. On the basis of the expression levels of 11 PRGs, Kaplan–Meier survival analysis was performed using two-sided log-rank tests with a threshold of *P* < 0.05. Then, using R package “glmnet”, least absolute shrinkage and selection operator (LASSO) Cox regression analysis was performed in order to select candidate PRGs and develop a pyroptosis-related prognostic gene model. The penalty parameter (λ) was decided by the minimum criteria and 7 PRGs were gained along with their coefficients in the end. The risk score formula was as follows:


$$Risk\;score=\sum_{i=1}^{n}\left(Expression_{i}\times Coefficient_{i}\right)$$

The cutoff of risk score in COAD from TCGA cohort is defined as the Youden’s index of receiver operating characteristic (ROC) curve for a 5-year survival and the risk score is maximum. According to the cutoff, they were classified into low-risk subgroup and high-risk subgroup. Principal component analysis (PCA) based on the 7 PRGs was performed for effective dimension reduction, pattern recognition, and exploratory visualization of COAD patients in subgroups. Additionally, with R packages “survival”, “survminer”, and “timeROC”, Kaplan–Meier survival analysis and time-dependent ROC analysis were applied to draw Kaplan–Meier curves, 1-year, 3-year, and 5-year overall survival (OS) ROC curves. Then, a GEO database (GSE17537) was served as validation cohorts. The risk score was then calculated by the same formula used for the TCGA cohort. The cutoff of risk score in COAD from GEO cohort is defined as the Youden’s index of ROC curve for 5-year survival and risk score. And patients were classified into two subgroups according to the cutoff. Additionally, 1-year, 3-year, and 5-year ROC curves were drawn.

### Establishment of a Predictive Nomogram

After assuring the patients in TCGA whose risk score and other clinical characteristics, including gender, age, tumor stage, T stage, N stage, and M stage, and whether prior malignancy or not is complete, 364 COAD patients remained. A protein–protein interaction (PPI) network of 51 PRGs was constructed by the Search Tool for the Retrieval of Interacting Genes (STRING) 11.0 and visualized in Cytoscape 3.8.2. Then, the CytoHubba plugin in Cytoscape was applied to determine the top 5 genes as hub genes according to their degree. The univariate and multivariate Cox regression analysis were conducted to testify whether these factors are concerned with the prognosis of COAD patients. On the basic of independent prognostic factors, R package “rms” and “survival” were employed to formulate a nomogram, which is used to individualize the survival probability for 1-year, 3-year, and 5-year OS. Then, concordance index (C-index), calibration curve, time-dependent ROC analysis, and decision curve analysis (DCA) were applied to evaluate the discrimination, calibration and clinical usefulness of nomogram.

### Functional Enrichment Analysis Based on Gene Model

The DEGs between low-risk score subgroup and high-risk score subgroup based on the median risk score were selected with a threshold of |log2FC| > 1 and FDR < 0.05. Subsequently, GO enrichment and KEGG pathway analysis were performed utilizing R package “clusterProfiler” based on these DEGs, with the criteria of |log2FC| > 1 and FDR < 0.05. Both of them were described by R package “ggplot2”.

### Association Between 7 Prognostic PRGs and Tumor Microenvironment

Tumor immune estimation resource (TIMER) database (https://cistrome.shinyapps.io/timer) was used to explore the correlation between 7 prognostic PRGs and immune infiltration. The “Gene” module of TIMER could realize visualization of the correlation between gene expression of COAD patients in TCGA cohort and the immune infiltration level of 6 immune cell types including B cells, CD4+ T cells, CD8+ T cells, neutrophils, macrophages, and dendritic cells. Additionally, TISIDB (http://cis.hku.hk/TISIDB/) was used to explore the correlation between 7 PRGs and immunoinhibitors for the treatment of COAD.

### Comparison of Immune Infiltration Between Subgroups

To characterize the immune TME between different risk subgroups based on the median risk score, CIBERSORT, an approach to characterize the immune cell composition of complex tissues based on their gene expression profiles, was utilized to evaluate the relative levels of the 22 immune cell phenotypes by using R package “CIBERSORT”.

### Immunohistochemical Staining of Candidate PRGs

The protein expression level of BTK (0.0825 mg/ml, HPA002028, Atlas Antibodies), GZMA (0.1114 mg/ml, HPA054134, Atlas Antibodies), EEF2K (CAB007818, abcam), NR1H2 (0.1556 mg/ml, HPA056838, Atlas Antibodies), SDHB (CAB009822, abcam) in COAD and normal tissue was verify by immunohistochemical staining, which obtained from Human Protein Atlas (HPA, https://www.proteinatlas.org/) database.

### Quantitative Realtime PCR

The 23-paired COAD tissue were collected from patients who underwent surgical resection for COAD at the Second Affiliated Hospital of Wenzhou Medical University (Wenzhou, China).The total RNA was extracted by using TRNzol Reagent and was reverse-transcribed with ReverTra Ace^®^ qPCR RT Master Mix with gDNA Remover (TOYOBO, JAPAN). All qPCR reactions were performed with QuantiNova SYBR Green RT-PCR Kit (QIAGEN, Germany) in 20 µl volume containing 10 µl 2× SYBR Green RT-PCR Master Mix, 1.4 µlof each 10 µM forward and reverse primer, 1 µl of cDNA sample, and nuclease-free water up to 20 µl. Amplification was carried out according to the following conditions: initial denaturation 95°C 2 min, followed by 40 cycles of denaturation 95°C 5 s, annealing 60°C 10 s. GAPDH was used as internal control. The relative expression of the gene was calculated by the 2^-△Ct method. The primers are listed in **Table 1**.

**Table 1:** List of primers.

Primer	Sequence
SDHB	FP: GACACCAACCTCAATAAGGTCTC RP : GGCTCAATGGATTTGTACTGTGC
CASP5	FP : TCACCTGCCTGCAAGGAATG RP : TCTTTTCGTCAACCACAGTGTAG
RIPK3	FP: ATGTCGTGCGTCAAGTTATGG RP : CGTAGCCCCACTTCCTATGTTG
NR1H2	FP: GTGGACTTCGCTAAGCAAGTG RP : ATGATCTCGATAGTGGATGCCT
EEF2K	FP: AACCTAACAAAAAGTGAGCGGT RP : GCCTTCTGGATTGCGTGCT
BTK	FP: TCCGAGAAGAGGTGAAGAGTC RP : AGAAGACGTAGAGAGGCCCTT
GZMA	FP: ATTCTTGGGGCTCACTCAATAAC RP : GGGTCATAGCATGGATAGGGAAA

## Results

### Identification of Differentially Expressed PRGs Between COAD and Normal Tissues

**Figure 1:**
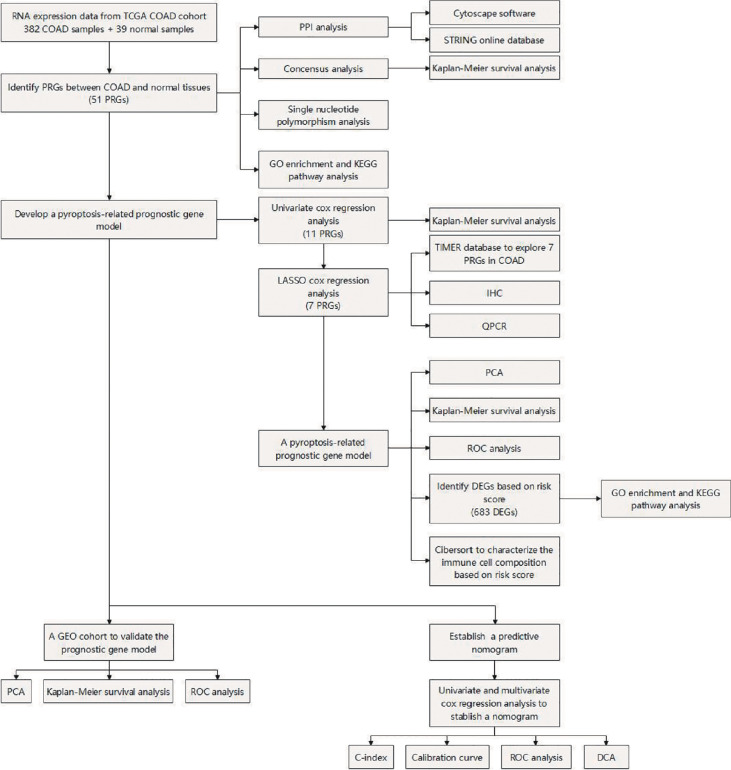
Flowchart of the study process.

**Figure 2:**
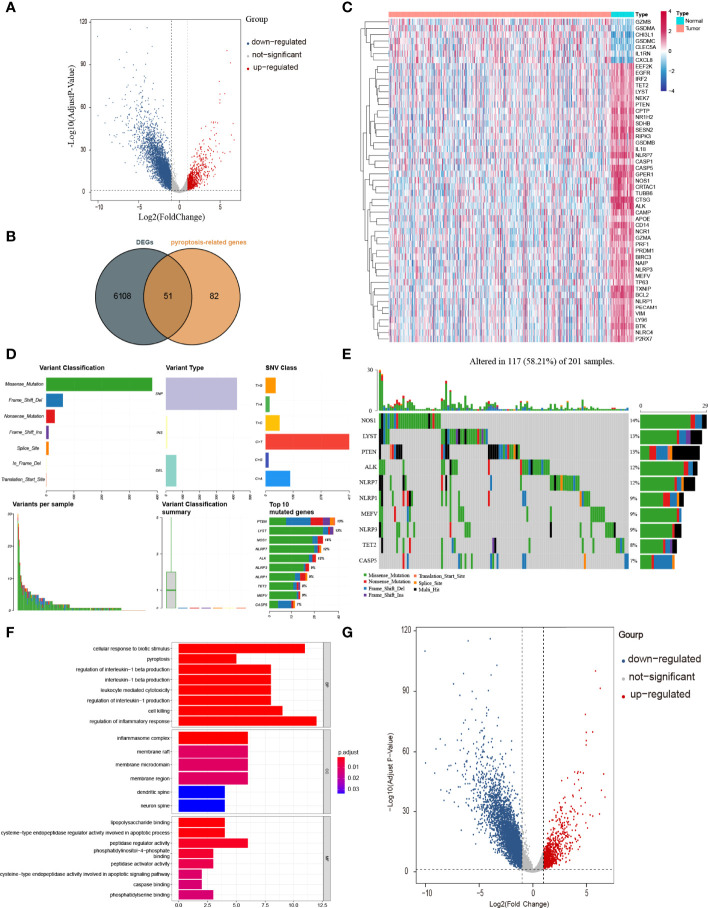
Identification of PRGs between COAD and normal tissues. **(A)** Volcano plot of gene expression in TCGA cohort. **(B)** Venn plot of DEGs identified in TCGA cohort and PRGs downloaded from GeneCards database. **(C)** Heatmap of 51 PRGs between 39 normal tissues and 382 COAD. **(D, E)** The mutation frequency and classification of 51 PRGs in COAD. **(F)** The top 30 significantly enriched GO terms in BP, CC, and MF. **(G)** The top most enriched KEGG pathways.

The workflow of our study is shown in **Figure 1**. To investigate the differentially expressed genes (DEGs) between colon adenocarcinoma (COAD) and normal tissues, we compared the raw data (counts) of gene expression between 39 normal tissues and 382 COAD from The Cancer Genome Atlas (TCGA) database with thresholds of |log2FC| > 1 and false discovery rate (FDR) < 0.05. A total of 6,159 DEGs were identified, in which the expression of 5,343 DEGs is decreased and the expression of 816 DEGs is increased in COAD compared with those in normal tissues (**Figure 2A**). After intersecting with 133 pyroptosis-related genes (PRGs) from the GeneCards database, a total of 51 PRGs among DEGs, containing 44 downregulated PRGs and 7 upregulated PRGs, remained (**Figure 2B**). And the expression level of these differentially expressed PRGs is shown in **Figure 2C**.

### Overview of Genetic Variation of 51 PRGs

To have a comprehensive insight into the incidence of somatic mutations of 51 PRGs, we performed single nucleotide polymorphism (SNP) analysis on COAD dataset from TCGA. As shown in **Figure 2D**, 117 of 201 (58.21%) COAD samples demonstrate genetic mutations, among which missense mutation is the most common variant classification. Additionally, SNP is the most common variant type, among which C > T ranks the top in the list of SNP classes. And the top 10 genes (NOS1, LYST, PTEN, ALK, NLRP7, NLRP1, MEFV, NLRP3, TET2, and CASP5) in mutation frequency rank were listed (**Figure 2E**).

### Functional Enrichment Analysis of 51 PRGs

To figure out the potential functions and pathways involved of 51 PRGs, Gene ontology (GO) enrichment analysis and Kyoto Encyclopaedia of Genes and Genomes (KEGG) pathway analysis were performed.

The GO results showed that these PRGs were enriched in pyroptosis-related and inflammatory-related terms, such as pyroptosis (*P* = 9 × 10^−11^), regulation of inflammatory response (*P* = 9 × 10^−9^) and inflammasome complex (*P* = 3 × 10^−12^; **Figure 2F**). KEGG analysis also showed that these PRGs were enriched in the cell death related pathway (**Figure 2G**).

### Development of a Pyroptosis-Related Prognostic Gene Model

**Figure 3:**
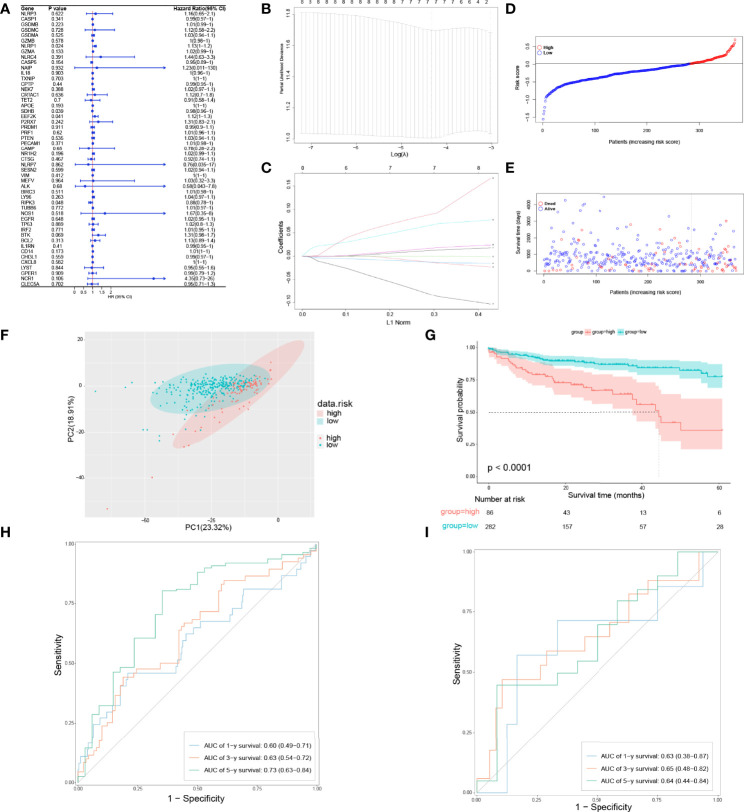
Development of a pyroptosis-related prognostic gene model. **(A)** Univariate Cox regression analysis of OS in TCGA cohort. **(B, C)** Gene selection by LASSO Cox regression analysis. **(D)** Distribution of patients based on the risk score. **(E)** The survival status for each patient (low-risk population: on the left side of the dotted line; high-risk population: on the right side of the dotted line). **(F)** PCA plot for COAD patients based on the risk score. **(G)** Survival analysis between low-risk and high-risk subgroups in COAD patients. **(H)** Time-dependent ROC analysis of the pyroptosis-related prognostic gene model in TCGA cohort. **(I)** Time-dependent ROC analysis of the pyroptosis-related prognostic gene model in validation set.

To figure out whether 51 PRGs are concerned with the prognosis of COAD patients, univariate Cox regression analysis and least absolute shrinkage and selection operator (LASSO) Cox regression analysis were used. Initially, univariate Cox regression analysis was applied for primary screening of survival-related genes. Based on the criteria of *P* < 0.2, 11 PRGs (APOE, BTK, CASP5, CD14, EEF2K, GZMA, NCR1, NLRP1, NR1H2, RIPK3, and SDHB) were selected for further analysis, among which 7 PRGs (BTK, CD14, EEF2K, GZMA, NCR1, NLRP1, and NR1H2) were associated with increased risk (HRs > 1), 3 PRGs (CASP5, RIPK3, SDHB) were connected with decreased risk (HRs < 1), and 1 PRG (APOE) is an exception with HRs = 1 (**Figure 3A**). Then, LASSO Cox regression analysis was performed on 11 genes screened by univariate Cox regression analysis (**Figure 3B**). According to optimum λ value, a pyroptosis-related prognostic gene model was developed on the basic of 7 PRGs (BTK, CASP5, EEF2K, GZMA, NR1H2, RIPK3, and SDHB, **Figure 3C**), whose coefficients are shown in **Table 2**. The risk score is calculated as follows: risk score = GZMA × 0.009700763 + CASP5 × -0.008832146 + SDHB × -0.010979061 × EEF2K × 0.058084338 + NR1H2 × 0.013626306 + RIPK3 × -0.069373213 + BTK × 0.080178474. In these 7 PRGs, 3 PRGs (CASP5, RIPK3, and SDHB) were protective factors, while 4 PRGs (BTK, EEF2K, GZMA, and NR1H2) were hazard factors, which was almost the same as the conclusion drawn from univariate Cox regression analysis. The cutoff of risk score in COAD from TCGA cohort was defined as the Youden’s index of receiver operating characteristic (ROC) curve for 5-year survival and risk score was maximum. Based on the cutoff = 0.014004, 368 COAD patients were separated into high-risk subgroup and low-risk subgroup (high risk: 86, low risk: 282, **Figure 3D**). Compared with the low-risk subgroup (median OS = 22 months), patients in the high-risk subgroup (median OS = 9 months) had higher mortality (**Figure 3E**, on the right side of the dotted line). These patients with different risk scores were explicitly separated into 2 clusters through principal component analysis (PCA, **Figure 3F**). Furthermore, 2 subgroups differed from each other significantly in OS (*P* < 0.001, **Figure 3G**). In order to assess the sensitivity and specificity of this pyroptosis-related prognostic gene model, time-dependent ROC analysis was adopted. The area under curve (AUC) in ROC was 0.60 for 1-year, 0.63 for 3-year, and 0.73 for 5-year survival, representing an efficient predictive efficacy (**Figure 3H**). The prognostic efficacy of the gene model was further verified in GSE17537. Like COAD patients in TCGA cohort, these patients in GEO cohort were divided into two subgroups based on the cutoff of risk score in COAD from two GEO cohorts. Time-dependent ROC analysis was applied to the prognostic gene model and indicated that our model has good prognostic effect with AUC = 0.63 for 1-year, 0.65 for 3-year, and 0.64 for 5-year survival (**Figure 3I**).

**Table 2:** Pyroptosis-related signature score.

Genes	Coefficients
GZMA	0.009700763
CASP5	-0.008832146
SDHB	-0.010979061
EEF2K	0.058084338
NR1H2	0.013626306
RIPK3	-0.069373213
BTK	0.080178474

### Establishment of a Predictive Nomogram

**Figure 4:**
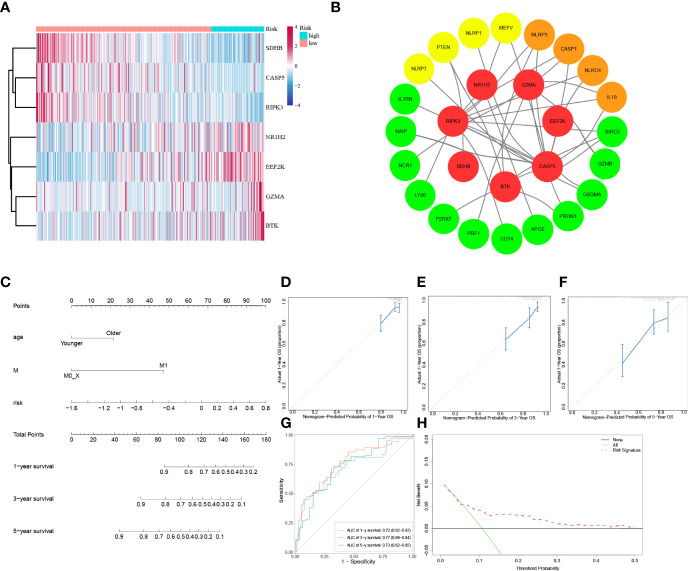
Establishment of a predictive nomogram. **(A)** Heatmap of 7 PRGs from prognostic gene model between low-risk and high-risk subgroups. **(B)** PPI network of 7 PRGs from prognostic gene model. **(C)** The nomogram based on the independent prognostic factors. **(D, E, F)** The calibration curves of the nomogram in 1-year, 3-year, and 5-year. **(G)** Time-dependent ROC curve analysis of the nomogram. **(H)** The DCA curve of the nomogram.

To explore whether the risk score derived from our pyroptosis-related prognostic gene model can be regarded as independent prognostic factor, we performed a Cox regression analysis in order to eliminate confounding factors (**Table 3**). Univariate analysis was performed to identify factors which might affect the survival of COAD patients from TCGA cohort, followed by multivariate analysis, which was controlled for potential confounders. Significantly, multivariate analysis confirmed that high-risk score was an independent risk factor for survival of COAD patients (HR = 3.62, 95% CI: 1.63–8.03, *P* = 0.002). Besides, a heatmap of the expression of 7 PRGs in COAD based on risk score was generated in **Figure 4A**. To further detect the interactions of 7 PRGs and the mechanisms of regulating COAD development, a protein–protein interaction (PPI) network with 51 nodes was constructed through the Search Tool for the Retrieval of Interacting Genes (STRING) online database (**Supplementary Figure 1**). Then the results were downloaded and analyzed in the Cytoscape software (**Supplementary Figure 2**). CASP1, IL18, CXCL8, NLRP3, and NLRC4 are regarded as the top 5 hub genes owing to their top degree in PPI network by applying CytoHubba plugin in Cytoscape. Furthermore, a PPI network was constructed with 7 PRGs (red: BTK, CASP5, EEF2K, GZMA, NR1H2, RIPK3, and SDHB) in our pyroptosis-related prognostic gene model, 4 hub genes (orange: CASP1, IL18, NLRP3, and NLRC4), 5 genes (yellow: PTEN, NLRP7, NLRP1, MEFV, and CASP5) in the top 10 genes in mutation frequency rank and other related proteins (green, **Figure 4B**). Clinical characteristics of patients are listed in the **Table 4**. Based on the relevant prognostic factors, a prognostic nomogram was established in order to effectively predict the prognosis of COAD patients (**Figure 4C**). With previous correlation analyses between predict effect and clinical characteristics along with risk score, Kaplan–Meier survival analysis was performed based on age (*P* = 0.02, **Supplementary Figure 3**) and M stage (*P* < 0.0001, **Supplementary Figure 4**). To evaluate this nomogram we built, concordance index (C-index) apt at estimating the predictive efficacy of single model and ROC analysis were adopted. The calibration curves of the nomogram in 1-year, 3-year, and 5-year showed a strong consistency between the observed and predicted values (**Figures 4D–F**). The consequence that C-index was 0.727 (0.688–0.766) and AUC in time-dependent ROC was 0.72 for 1-year, 0.77 for 3-year, and 0.73 for 5-year survival indicated that our nomogram did well in predicting the prognosis of COAD patients (**Figure 4G**). In addition, the decision curve analysis (DCA) curve suggested this nomogram had excellent clinical prospect in predicting the OS of COAD patients (**Figure 4H**). To sum up, this predictive nomogram had an ideal capacity for predicting 1-year, 3-year, and 5-year OS of COAD patients.

**Table 3:** Univariate and multivariate Cox regression analysis for COAD with clinicopathological factors in TCGA cohort.

Characteristics	Univariate Cox regression analysis		Multivariate Cox regression analysis	
	Hazard ratio (95% CI)	*P* value	Hazard ratio	*P* value
			(95% CI)	
Age	0.54 (0.32–0.91)	0.022	0.44 (0.26–0.76)	0.003
Gender	1.16 (0.73–1.85)	0.529		
M	4.7 (2.88–7.66)	<0.001	2.83 (1.58–5.08)	<0.001
N	0.37 (0.23–0.59)	<0.001	1.49 (0.44–5.05)	0.52
Prior_malignancy	1.11 (0.58–2.11)	0.753		
Risk	4.79 (2.26–10.15)	<0.001	3.62 (1.63–8.03)	0.002
T	2.93 (1.18–7.29)	0.021	1.48 (0.56–3.88)	0.43
Tumor_stage	3.13 (1.92–5.1)	<0.001	3.12 (0.79–12.35)	0.104

**Table 4:** Baseline characteristics of COAD patients.

Characteristics	Low risk(*n* = 278)	High risk(*n* = 86)
Gender		
Male	148	44
Female	130	42
Age		
Younger (<65)	105	31
Older (≥65)	173	55
Tumor_stage		
Stage I_II	168	41
Stage III_IV	110	45
T		
T1_2	58	13
T3_4	220	73
N		
N0	173	43
N+	105	43
M		
M0_X	244	67
M1	34	19
Prior_malignancy		
Yes	37	13
No	241	73
Survival status		
Alive	237	54
Dead	41	32

### Functional Enrichment Analysis Based on Gene Model

To deepen the understanding of distinction in the gene functions and pathways between high-risk subgroup and low-risk subgroup in COAD patients from TCGA cohort classified by the prognostic gene model, a total of 683 DEGs with a threshold of |log2FC| > 1 and FDR < 0.05 were identified, among which 635 DEGs are upregulated and 48 DEGs are downregulated in the high-risk subgroup compared with that in the low-risk subgroup and GO enrichment analysis as well as KEGG pathway analysis were carried out.

**Figure 5:**
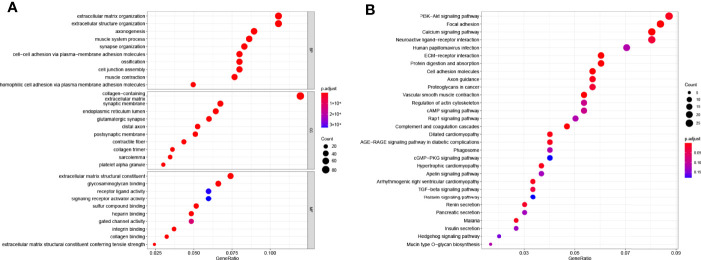
Functional enrichment analysis based on gene model. **(A)** The top 30 significantly enriched GO terms of DEGs gained from the comparison in low-risk and high-risk subgroups in BP, CC, and MF. **(B)** The top most enriched KEGG pathways.

The GO results showed that these DEGs were enriched in cell junction and signal transduction related terms (**Figure 5A**). The KEGG results showed that these DEGs were closely enriched in PI3K-AKT signaling pathway (*P* = 0.0006) and focal adhesion (*P* = 8 × 10^−8^, **Figure 5B**).

### Association Between 7 Prognostic PRGs and Tumor Microenvironment

**Figure 6:**
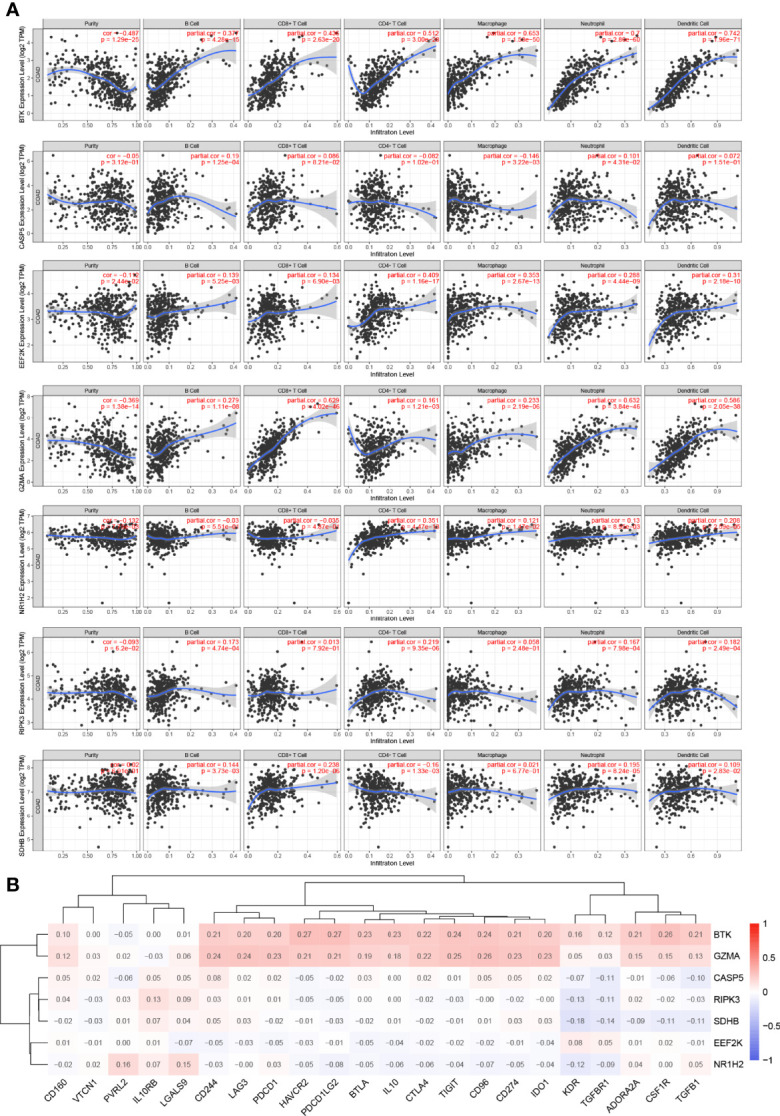
Association between 7 prognostic PRGs and tumor microenvironment. **(A)** The association between the abundance of immune cells and the expression of BTK, CASP5, EEF2K, GZMA, NR1H2, RIPK3, and SDHB in COAD. **(B)** The relationships between 7 PRGs and immunoinhibitors.

It is widely known that pyroptosis has a close relation with the tumor microenvironment. To clarify the correlation between 7 PRGs (BTK, CASP5, EEF2K, GZMA, NR1H2, RIPK3, and SDHB) derived from LASSO Cox regression analysis and immune infiltration in COAD, we applied Tumor Immune Estimation Resource (TIMER) database on these genes. Based on the assumption that a correlation coefficient >0.3 is viewed as a strong correlation, TIMER database was employed to demonstrate the purity-corrected partial Spearman’s rho value and statistical significance (**Figure 6A**). BTK was associated with the infiltration of B cell (*P* = 4.28 × 10^−15^), CD8+ T cell (*P* = 2.63 × 10^−20^), CD4+ T cell (*P* = 3 × 10^−28^), macrophage (*P* = 1.53 × 10^−50^, neutrophil (*P* = 2.86 × 10^−60^), and dendritic cell (*P* = 1.96 × 10^−71^) positively. Besides, it showed a positive association between EEF2K expression and the abundance of CD4+ T cell (*P* = 1.16 × 10^−17^, macrophage (*P* = 2.67 × 10^−13^), and dendritic cell (*P* = 2.18 × 10^−10^). There existed a positive correlation between the expression of GZMA and the abundance of CD8+ T cell (*P* = 4.02 × 10^−46^), neutrophil (*P* = 3.84 × 10^−46^), and dendritic cell (*P* = 2.05 × 10^−38^). Additionally, the expression of NR1H2 had a positive correlation with the abundance of CD4+ T cell (*P* = 4.47 × 10^−13^). Evidently, there existed a strong correlation between 7 PRGs and tumor microenvironment in COAD. Furthermore, we did some researches on seeking relationships between 7 PRGs and immune inhibitors from TISIDB. It figured out that 7 PRGs, especially BTK and GZMA, were strongly linked with immune inhibitors, indicating a brilliant treatment effect (**Figure 6B**).

### Comparison of Immune Infiltration Between Subgroups

**Figure 7:**
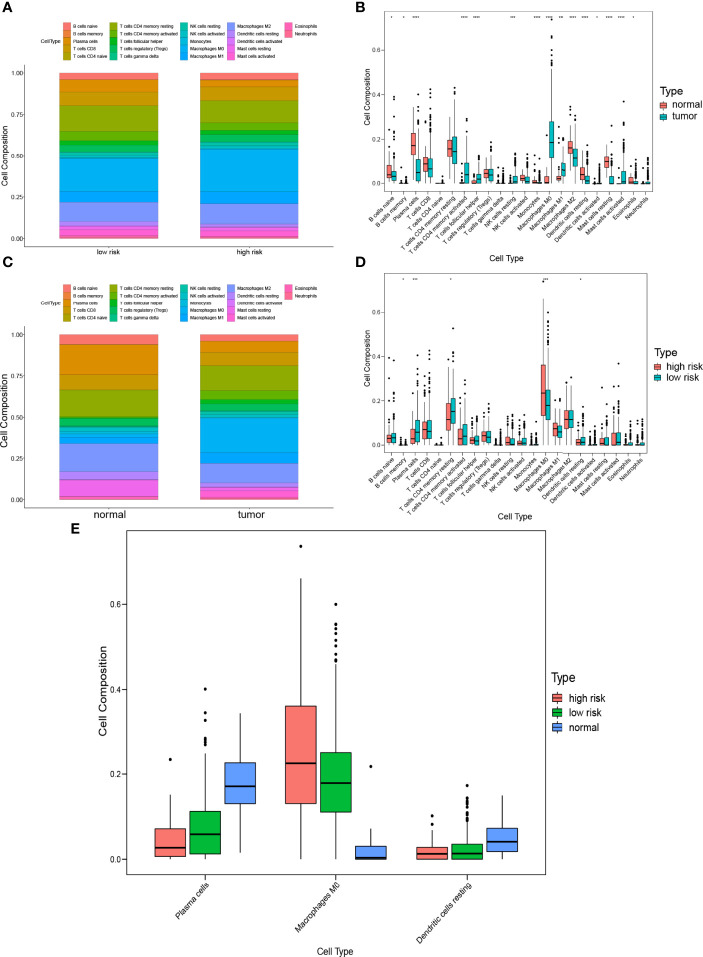
Comparison of immune infiltration between subgroups. **(A)** Proportions of immune cells between normal tissue and COAD. **(B)** Violin plot of the differentiation of immune cells between normal tissue and COAD. **(C)** Proportions of immune cells in low-risk and high-risk subgroups. **(D)** Violin plot of the differentiation of immune cells in low-risk and high-risk subgroups. **(E)** Violin plot of the differentiation of immune cells in normal tissue, low-risk and high-risk subgroups.

To characterize the difference of the immune infiltration between high-risk subgroup and low-risk subgroup in TCGA COAD cohort according to the gene model, the CIBERSORT method was applied. The distribution of 22 immune cells in normal tissue and COAD from TCGA cohort was demonstrated in **Figure 7A**. Data suggested that the abundance of B cells naive, B cells memory, plasma cells, monocytes, macrophages M2, dendritic cells resting, mast cells resting, and eosinophils in tumor was lower than that in normal tissue, while the abundance of T cells CD4 memory activated, T cells follicular helper, NK cells resting, macrophages M0, macrophages M1, dendritic cells activated, and mast cells activated in tumor was higher than that in normal tissue (**Figure 7B**). Besides, the distribution of 22 immune cells in low-risk subgroup and high-risk subgroup from TCGA cohort was demonstrated in **Figure 7C**. The result indicated that the abundance of plasma cells, T cells CD4 memory resting, and dendritic cells resting in the high-risk subgroup were lower than that in the low-risk subgroup, while the abundance of B cells memory and macrophages M0 in the high-risk subgroup was higher than that in the low-risk subgroup (**Figure 7D**). The result of two comparisons was summarized in **Table 5**. There were consistent changes in the abundance of 3 immune cells (plasma cells, macrophages M0, and dendritic cells resting) in two comparisons. Specifically, the abundance of plasma cells and dendritic cells resting decreases while that of macrophages M0 increases from normal tissue to low-risk subgroup to high-risk subgroup in TCGA cohort (**Figure 7E**).

**Table 5:** The difference of the immune infiltration in normal tissues, low-risk subgroup and high-risk subgroup.

Cell type	The abundance in tumor compared with that in normal tissue	The abundance in high-risk subgroup compared with that in low-risk subgroup	Consistency
B cells naive	↓		
B cells memory	↓	↑	
Plasma cells	↓	↓	↓
T cells CD8			
T cells CD4 naive			
T cells CD4 memory resting		↓	
T cells CD4 memory activated	↑		
T cells follicular helper	↑		
T cells regulatory (Tregs)			
T cells gamma delta			
NK cells resting	↑		
NK cells activated			
Monocytes	↓		
Macrophages M0	↑	↑	↑
Macrophages M1	↑		
Macrophages M2	↓		
Dendritic cells resting	↓	↓	↓
Dendritic cells activated	↑		
Mast cells resting	↓		
Mast cells activated	↑		
Eosinophils	↓		
Neutrophils			

### Expression Level of 7 Prognostic PRGs Between COAD and Normal Tissues

**Figure 8:**
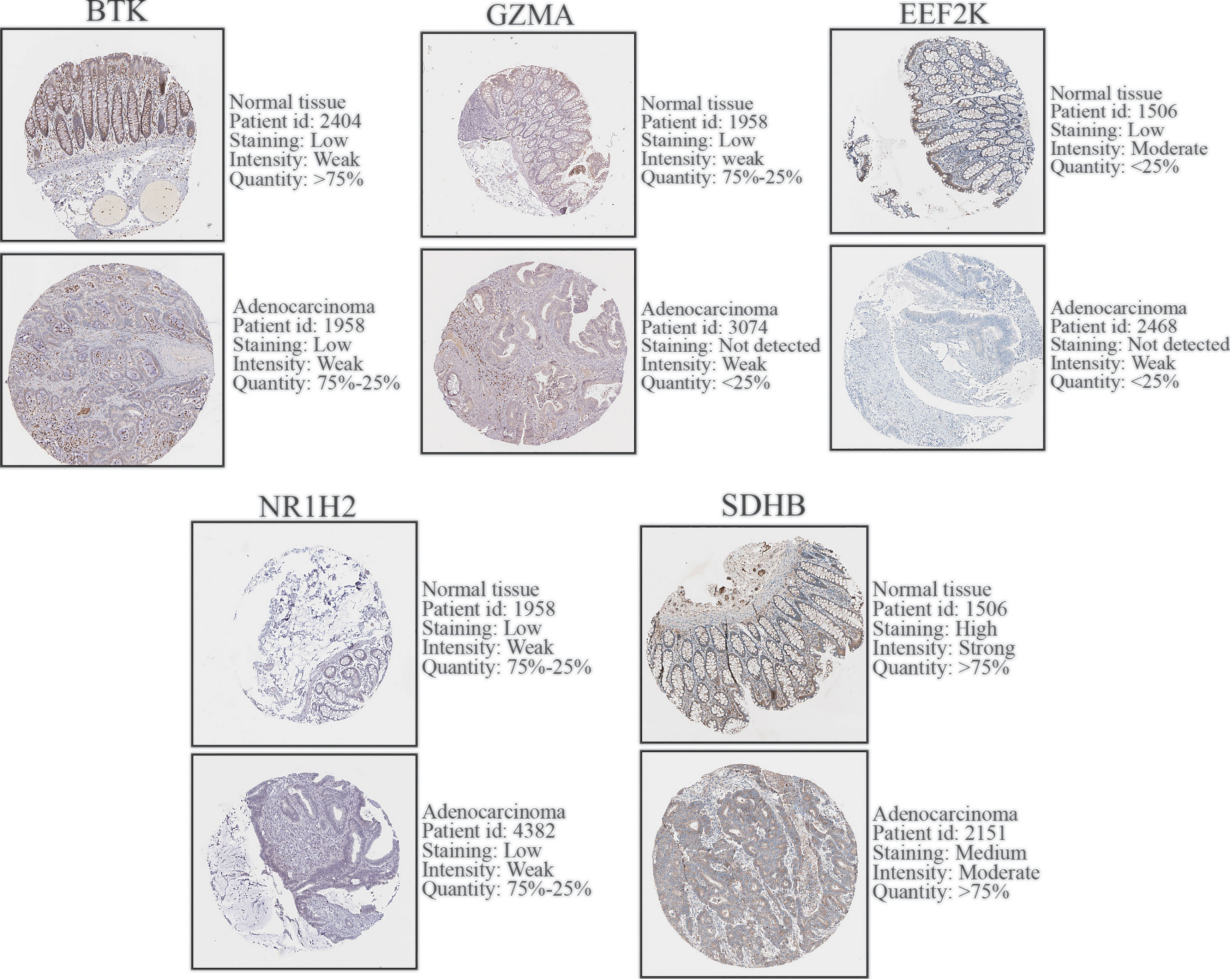
Immunohistochemical staining and instructions of candidate genes in COAD tissues and normal tissues in the HPA database.

**Figure 9:**
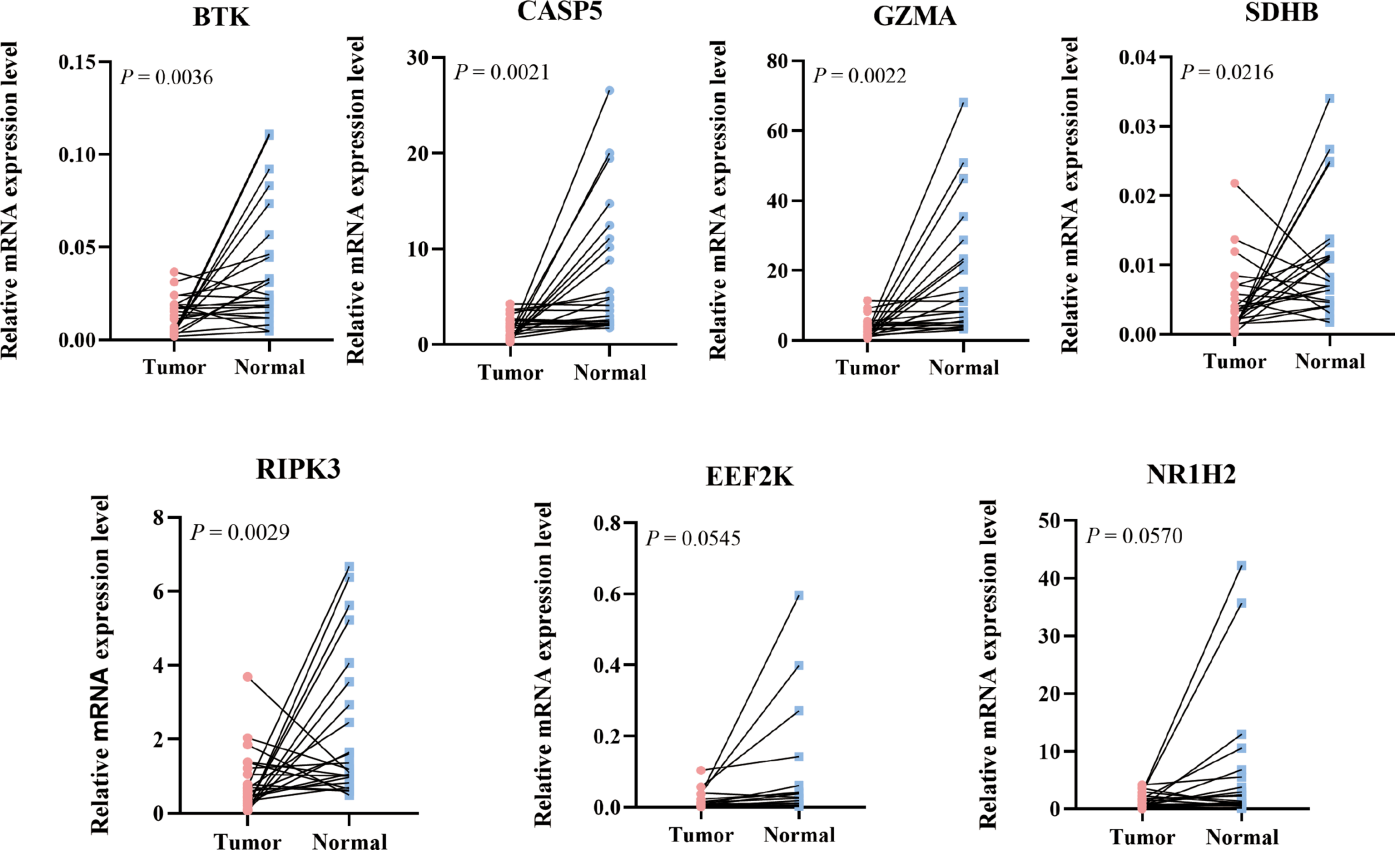
Relative mRNA expression level of 7 PRGs in COAD and adjacent normal tissues detected by qPCR.

To further validate the expression level of the 7 prognostic PRGs, immunohistochemical staining results for SDHB, GZMA, BTK, EEF2K, and NR1H2 were obtained from the HPA database, and qPCR was performed on 23 paired colon tumor and adjacent tissues. The results from HPA database showed that the immunohistochemical staining intensity of SDHB, GZMA, BTK, EEF2K, and NR1H2 in glandular cells of normal tissues was stronger than tumor cells, demonstrating that these genes were significantly expressed in normal colon tissues than in tumor tissues (**Figure 8**). qPCR results showed that the other 5 prognostic PRGs, except EEF2K and NR1H2, were significantly highly expressed in normal tissues (**Figure 9**).

## Discussion

CRC is a collection of several tumors, including rectum adenocarcinoma (READ), COAD, and other subtypes. In fact, prognostic properties and patterns differ from different subtypes of cancer, especially READ and COAD, which differ significantly in tumor progression, tumor microenvironment, and clinical treatment (21–26). Therefore, we concentrated on COAD, the subtypes of CRC alone, expecting to get more accurate understanding. In this study, we collected gene expression data and clinical information of COAD from TCGA and GEO database. A total of 51 PRGs were identified, of which a prognostic prediction model containing 7 RPGs was constructed with high predictive accuracy by LASSO analysis. The clinical characteristics and risk factors were integrated by nomogram, and the prognostic accuracy of the nomogram was confirmed by the ROC curve and calibration plots. GO and KEGG analyses indicated that the 51 PRGs were associated with pyroptosis, immune response, and several cell death-related pathways. The molecular alteration in the high- or low-risk group was closely associated with intercellular signal transduction. The results of IHC from HPA database and qPCR validated the expression level of the 7 PRGs in COAD. Taken together, these results strongly implied the critical roles of pyroptosis in COAD.

In recent years, several PRGs signature models for prognosis predication of cancers have been established. Ye et al. provided a novel gene signature including 7 PRGs (AIM2, PLCG1, ELANE, PJVK, CASP3, CASP6, and GSDMA) for predicting the prognosis of ovarian cancer (OC) patients and laid a foundation for further studies of the relationships between PRGs and immunity in OC (20). Lin et al. not only constructed a pyroptosis-related prognostic predication model involving 5 PRGs (CASP6, NLRP7, NOD1, NLRP1, and NLRP2) in lung adenocarcinoma, but also established a network of mRNA–miRNA–lncRNA closely relating to PRGs (27). Zhou et al. constructed a new prognosis predication model based on 8 risk-related PRGs (GPX4, GSDME, GZMA, GZMB, IL1B, NOD1, PRKACA, and TNF) in cervical cancer, which had good predictive ability (AUC = 0.794) (28). In colon cancer, however, similar studies have been limited. Song et al. previously identified two distinct molecular subtypes based on PRGs in COAD (29). Nevertheless, only 48 PRGs from MSigDB Team (REACTOME_PYROPTOSIS) were included in their study and the DEGs were not validated in their own cohort, showing obvious limitations. In our study, we considered more than 100 PRGs from GeneCards database and the DEGs in the final model were validated in our cohort, which partly avoided possible deviation.

The prognostic model proposed in the present study was composed of seven PRGs (BTK, CASP5, GZMA, SDHB, RIPK3, EEF2K, and NR1H2). Of them, BTK, CASP5, GZMA, SDHB, and RIPK3 were considered to be the key genes in our PRGs signature, which were further verified by qPCR in our own cohort. Bruton tyrosine kinase (BTK) is a crucial signaling molecule downstream of B cell receptor (BCR) and a previous study indicated that BTK regulates a step in the NLRP3 inflammasome activation and activates caspase-1 through ASC-mediated junctional proteins to promote the maturation and release of IL-1β and IL-18 and to promote pyroptosis (30). CASP5, a member of the cysteine-aspartic acid protease (caspase) family, can be activated by saturated fatty acids in human monocytes, triggering IL-1β and IL-18 release, which are known to be pyroptosis promoters (31). Granzyme A (GZMA) is the most abundant protease present in cytotoxic granules and is reported as the dominant mediator of toxicity *in vitro* (32). As GZMA from cytotoxic lymphocytes, it cleaves GSDMB to trigger pyroptosis in target cells (33). What is more, the concentrations of GZMA in patients with ovarian cancer were substantially increased in comparison to that in patients with ovarian cystadenomas or ovarian teratomas (34), which is similar to our results that the high expression of GZMA was connected with poor OS of COAD patients. Succinate dehydrogenase complex subunit B (SDHB), a subunit of SDH family located on the inner membrane of the mitochondria, plays a vital role in the respiratory chain and SDH oxidation promotes mitochondrial production of ROS (35), which mediates the proinflammation and pro‐pyroptosis signals (36). Receptor interacting serine/threonine kinase 3 (RIPK3) has also been proved to be a key regulator of necroptosis (37).

To deepen the understanding of distinction between high-risk subgroup and low-risk subgroup in TCGA cohort, functional enrichment analyses were performed and the results showed that these DEGs were related to many classical tumor-related pathways, such as PI3K−Akt signaling pathway. The significant correlation between 7 PRGs and several immune cells, including B cells, macrophages, dendritic cells, and T cells, was found by following TIMER analysis, which also indicated the importance of pyroptosis in tumor immune microenvironment (TIME). Furthermore, the CIBERSORT analysis reveals that plasma cells, resting dendritic cells, and eosinophils upregulated while M0 macrophages downregulated in the high-risk score subgroup. In fact, plasma cells could predict patients’ survival by using a variety of markers including CD138 and IGKC (38). Additionally, the importance of macrophages, resting dendritic cells, and eosinophils in cancer immunotherapy has been reported (39–41). In our study, plasma cells and resting dendritic cells benefit OS of COAD patients while M0 macrophages and eosinophils do harm to OS of COAD patients. Overall, besides prognostic gene model, the immune cells in TIME are promising prognostic biomarker candidates in COAD. Additionally, the correlation exploration in TISIDB indicates that these 7 PRGs, especially BTK and GZMA, are expected to become new targets for gene immunotherapy because they have a close relationship with immunoinhibitors such as PD-L1 (CD274) and CTLA-4 (CTLA4). It is reported that dual CTLA-4 and PD-L1 blockade has an excellent performance in inhibiting tumor growth and liver metastasis in a highly aggressive orthotopic mouse model of colon cancer (42).

Our study has several limitations. First, in TCGA cohort, the number of normal samples was much smaller than tumor samples, which might result in a statistical deviation in the DEGs analysis. Second, although the prognostic predictive effect was well established, the underlying mechanism of how these PRGs modulate the process of COAD is still unclear. Lastly, although the PRGs that were identified in our study may not be complete, there is a great significance in the improvement of the PRG prognostic model in COAD.

## Conclusion

In this study, we identified a pyroptosis-related prognostic gene signature, which is able to predict the prognosis of COAD patients and is associated with immune infiltration in COAD. These findings may further our understanding of TME and shed light on the development of novel prognostic biomarkers and therapeutic targets in COAD.

## Data Availability Statement

The datasets presented in this study can be found in online repositories. The names of the repository/repositories and accession number(s) can be found in the article/**Supplementary Material**.

## Ethics Statement

The studies involving human participants were reviewed and approved by Medical Ethics Committee of the Second Affiliated Hospital of Wenzhou Medical University. The patients/participants provided their written informed consent to participate in this study.

## Author Contributions

KL, XX, and XS designed and directed all the research. ZC, ZH, and HN performed the RNA−seq analyses. ZC, ZH, JF, and YZ drafted the manuscript. The RNA extraction, reverse transcription, and qPCR were performed by HZ and HN. JF and YC performed statistical analysis. JZ and YZ participated in the revision of the paper. All authors reviewed the manuscript. ZC, ZH, and HN contributed equally to this work. All authors read and approved the final manuscript.

## Funding

The work was supported by Key Laboratory of Tumor-Related Pathogens and Immunity of Wenzhou City; Key R&D program of Science Technology Department of Zhejiang Province [2020C03029].

## Conflict of Interest

The authors declare that the research was conducted in the absence of any commercial or financial relationships that could be construed as a potential conflict of interest. 

## Publisher’s Note

All claims expressed in this article are solely those of the authors and do not necessarily represent those of their affiliated organizations, or those of the publisher, the editors and the reviewers. Any product that may be evaluated in this article, or claim that may be made by its manufacturer, is not guaranteed or endorsed by the publisher.

## Supplementary Material

Supplementary Figure 1 PPI network of 51 PRGs in STRING.

Supplementary Figure 2 PPI network of 51 PRGs in Cytoscape.

Supplementary Figure 3 Survival analysis based on age in COAD patients.

Supplementary Figure 4 Survival analysis based on M stage in COAD patients.
