# Supplementary figures and images for: A Novel Pyroptosis-Related Gene Signature for Predicting the Prognosis and the Associated Immune Infiltration in Colon Adenocarcinoma
Source: Front Oncol. 2022 Jul 14;12:904464. doi: 10.3389/fonc.2022.904464 (PMC9330598; doi:10.3389/fonc.2022.904464)

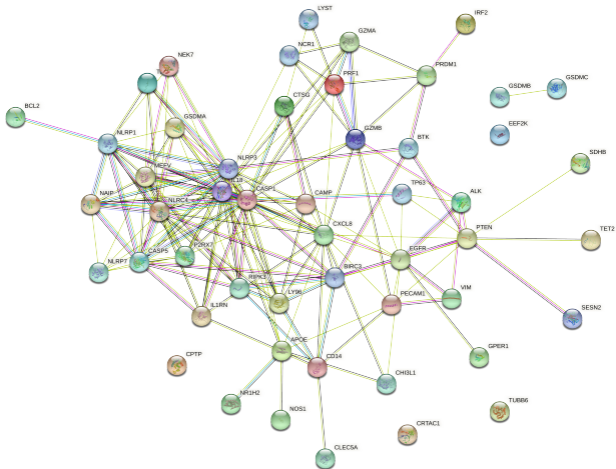

Supplement: Supplementary Figure 1 — PPI network of 51 PRGs in STRING. [file DataSheet_1.pdf]

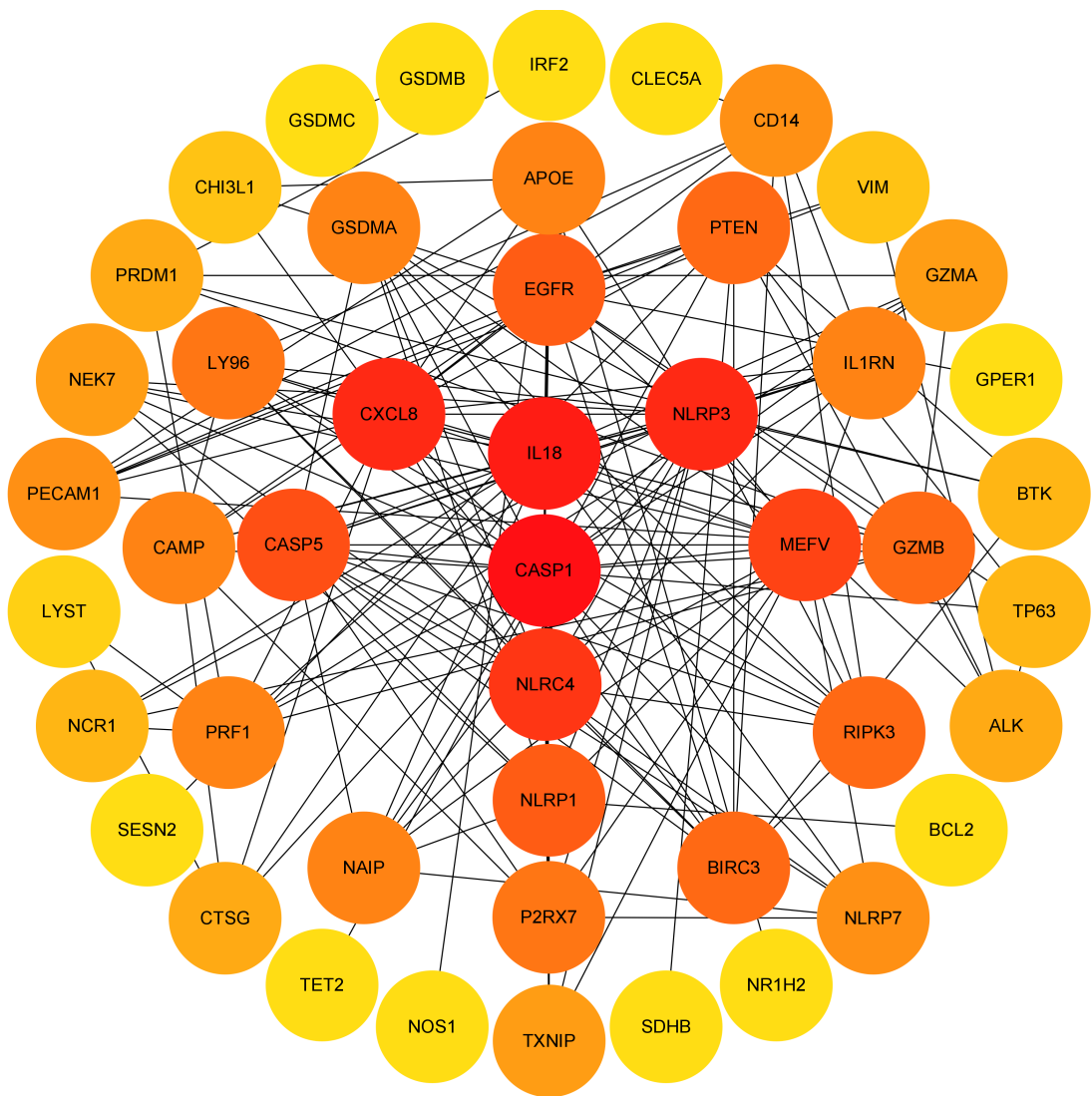

Supplement: Supplementary Figure 2 — PPI network of 51 PRGs in Cytoscape. [file DataSheet_2.pdf]

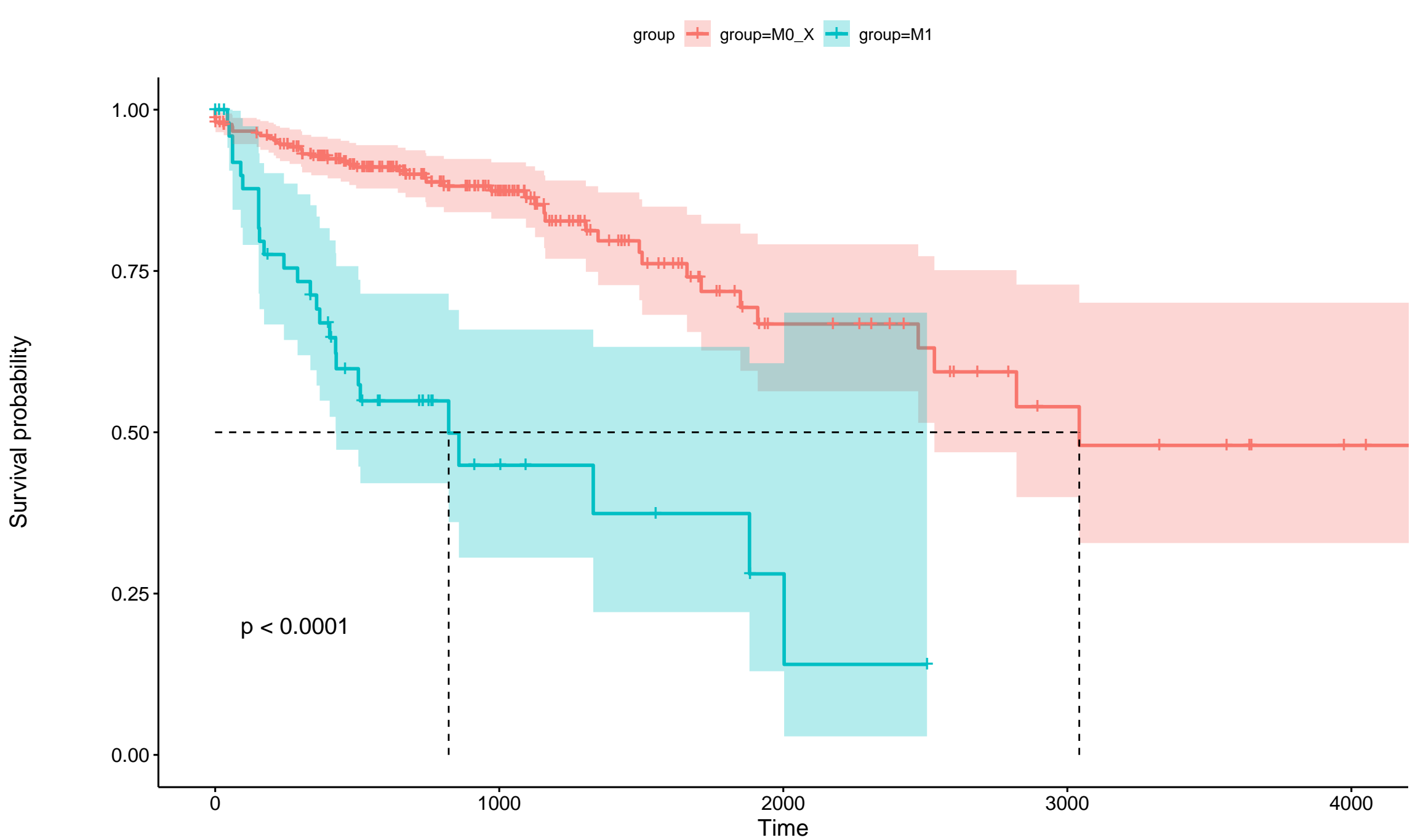

Number at risk

group=M0\_X

311

107

23

9

3

group=M1

53

8

2

0

0

Supplement: Supplementary Figure 4 — Survival analysis based on M stage in COAD patients. [file DataSheet_4.pdf]
