# Supplementary material for: A Novel Pyroptosis-Related Gene Signature for Predicting the Prognosis and the Associated Immune Infiltration in Colon Adenocarcinoma
Source: Front Oncol. 2022 Jul 14;12:904464. doi: 10.3389/fonc.2022.904464 (PMC9330598; doi:10.3389/fonc.2022.904464)
Supplement: Supplementary Figure 3 — Survival analysis based on age in COAD patients. [file DataSheet_3.pdf]

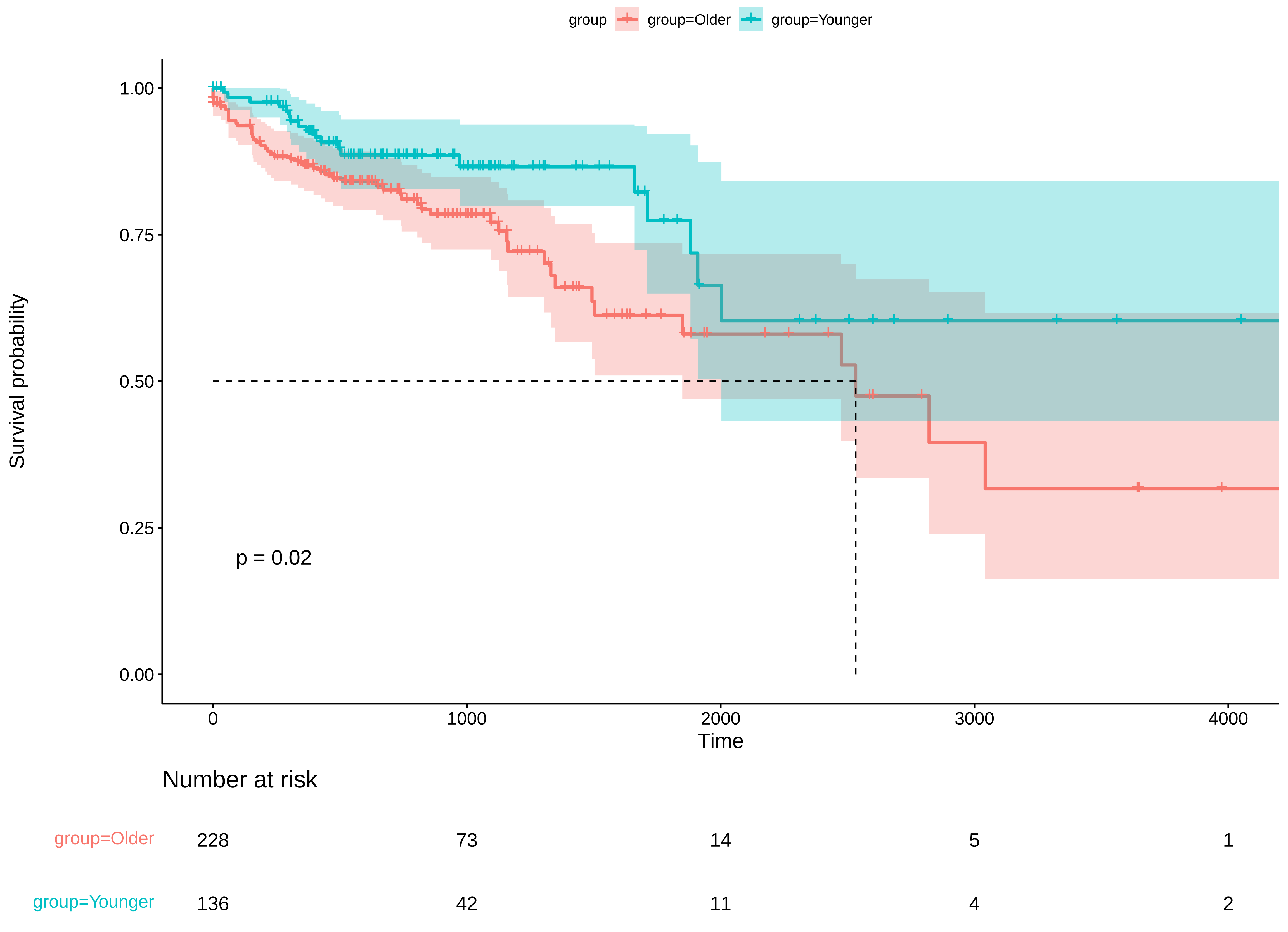

group
group=Older
group=Younger
Survival probability
1.00
0.75
0.50
0.25
0.00
p = 0.02
0
1000
2000
3000
4000
Time
Number at risk
group=Older 228 73 14 5 1
group=Younger 136 42 11 4 2
